# Factors associated with school absenteeism due to difficulty awakening: a two-year prospective cohort study of Japanese adolescents

**DOI:** 10.1265/ehpm.25-00290

**Published:** 2025-11-15

**Authors:** Yuichiro Otsuka, Mikiko Tokiya, Isao Saitoh, Osamu Itani, Yoshitaka Kaneita

**Affiliations:** 1 Division of Public Health, Department of Social Medicine, Nihon University School of Medicine, Tokyo, Japan; 2 Department of Social and Environmental Medicine, Faculty of Medicine, Saga University, Saga, Japan; 3 Department of Public Health and Epidemiology, Faculty of Medicine, Oita University, Oita, Japan; 4 Department of Public Health, International University of Health and Welfare, Chiba, Japan

**Keywords:** Absenteeism, Adolescent, Risk factors, Sleep–wake disorders, Transportation

## Abstract

**Background:**

Difficulty awakening is a common concern among adolescents and contributes to school absenteeism. Although cross-sectional studies suggest that commuting times, internet usage, and sleep disturbances are associated with school attendance problems, few have specifically focused on adolescents. We aimed to examine the factors contributing to school absenteeism due to difficulty awakening in Japanese high school students.

**Methods:**

In this longitudinal cohort study, data were prospectively collected between 2016 and 2018 from 54 high schools in a prefecture of Western Japan. Tenth-grade students (n = 6,121) without tardiness (n = 5,812) or absences (n = 5,946) at baseline were recruited. The outcome of interest was school absenteeism due to difficulty awakening, which included both tardiness and absences (≥2 days/month). Cox proportional hazards models were used to assess the associations between commuting time and lifestyle factors after adjusting for confounders.

**Results:**

The incidence rates of tardiness and absences due to difficulty awakening were 19.3 and 9.6 per 1,000 person-years, respectively. Common risk factors for absenteeism included prolonged internet usage (≥5 h) and dissatisfaction with school. Protective factors for school tardiness included study time and participation in extracurricular sports activities (both ≥2 h). Long commuting time (≥1 h) was associated with a higher risk of school absences.

**Conclusions:**

Long commuting times, prolonged internet usage, and poor school satisfaction increased the risk of school absenteeism due to difficulty awakening. Promoting academic engagement and extracurricular activities may help reduce absenteeism. Interventions that increase school satisfaction, such as providing learning opportunities outside of school, supporting extracurricular activities, and improving the school environment, may be effective prevention strategies.

**Supplementary information:**

The online version contains supplementary material available at https://doi.org/10.1265/ehpm.25-00290.

## Introduction

Sleep plays a crucial role in adolescent health, influencing physical, cognitive, and psychological well-being [1, 2]. Adolescents typically experience a delayed circadian phase, reduced sleep duration on school nights, and changes in sleep architecture when compared with other age groups [3]. Pubertal shifts in circadian regulation contribute to later sleep onset and wake times [3]. This pronounced shift toward a late chronotype during adolescence contrasts with the earlier sleep patterns commonly observed in adulthood [4].

Globally, approximately 30–70% of adolescents experience sleep-related concerns, including insufficient, irregular, non-restorative, or disrupted sleep, in addition to delayed sleep onset [5–8]. Meta-analyses demonstrate that millions of adolescents worldwide frequently experience inadequate sleep (<8 h), specifically on school nights [6]. Difficulty awakening in the morning is a common sleep-related issue among adolescents, with prevalence rates of 53.7% [9], 66.9% [10], and 76.2% [11] among those aged 16 in Sweden, 13–19 in Italy, and 12–18 years in Portugal, respectively. Factors associated with difficulty awakening include short sleep duration [12] and insomnia [13]; substance use, including alcohol consumption and smoking [13]; lack of participation in extracurricular activities [13]; reduced motivation to attend school [14]; use of electronic devices before bedtime [10]; and poor mental health [13].

Persistent morning awakening difficulties can disrupt academic adjustment and increase absenteeism, with considerable consequences, including academic underachievement and social challenges [15]. Poor academic adjustment and absenteeism represent substantial public health concerns for adolescents. Programme for International Student Assessment (PISA) data from 2012, 2015, and 2018 were used to assess school absenteeism, defined as tardiness or absence for >1 day per 2 weeks prior to the PISA test. Among students in Germany, Japan, Sweden, and the UK, the prevalence ranged from 1.5% in Japan to 24.4% in the UK [16]. Chronic absenteeism among public school students in the USA increased by 13.5% from 2018–2019 to 2021–2022, reflecting a 91% rise [17].

School absenteeism is influenced by various factors, including individual, parental, familial, peer, school, and community variables [18]. A recent meta-analytic review identified physical and mental health problems, substance abuse, antisocial behavior, school-related issues, low parental involvement, and ineffective family systems as important contributors to school absenteeism [19]. Moreover, sleep disorders, including difficulty awakening, are recognized as key factors in school refusal [20, 21].

A national, cross-sectional study of Japanese adolescents found that 15.5% of boys and 14.4% of girls were tardy for school for >1 day owing to difficulty awakening. Additionally, 5.6% of boys and 5.9% of girls were absent for >1 day owing to the same concern [13]. Research indicates that children with insomnia, parasomnia, or daytime somnolence are more prone to school refusal, particularly those with anxiety disorders [22]. These findings emphasize the importance of understanding the connection between sleep problems and school refusal [22].

Our previous study showed that extended commuting times for high school students are positively associated with anxiety disorders and depressive symptoms, regardless of sleep duration [23]. A cross-sectional study of Thai students aged 9–18 years revealed that long commuting times and distances are negatively associated with physical and mental health [24].

Although several studies have examined the association between commuting times and attendance issues among workers [25], only a few have focused on adolescents. Data collected by the Baltimore Consortium for Education and Research revealed that longer commutes are correlated with increased school absences, particularly during the transition from middle to high school [26]. Most studies to date have been cross-sectional, leaving a gap in research regarding factors contributing to school attendance problems due to difficulty awakening, particularly in longitudinal designs.

Thus, in the present study, we focused on the association between commuting times and school absenteeism due to difficulty awakening. Specifically, we aimed to: (1) identify risk factors for school tardiness and absences due to difficulty awakening and (2) assess the impact of commuting times on these outcomes using longitudinal data from high schools in a Japanese prefecture. We hypothesized that our findings would provide valuable insights for educational institutions in making school selection decisions for adolescents and implementing appropriate modifications to the school environment.

## Methods

### Ethical approval and informed consent

This study was approved by the Ethics Committee of Oita University, Faculty of Medicine, on November 30, 2015 (approval no. 932) and Nihon University School of Medicine on January 24, 2024 (approval no. 2024-22). It was conducted in accordance with the principles of the Declaration of Helsinki and STROBE reporting guidelines. Written informed consent was obtained from the adolescents and their parents or legal guardians for participation in the study, inclusion in the analysis, and publication of de-identified data.

### Participants and procedure

This study included data from a longitudinal survey of factors influencing lifestyle changes among high school students conducted by Oita University. The survey was based on a 2-year longitudinal observational study targeting 10th-grade students. Initiated in 2016, the survey targeted 54 full-time high schools in a prefecture of Western Japan. Invitations to participate in the survey were extended to the principals of these schools, and consent to participate was obtained from 40 principals (32 public schools/8 private schools) (10th-grade students: aged 15–16 years, n = 7,186). In 2016, surveys were distributed to students in the 10th grade, and in 2018, to students in the 12th grade. For each survey, consent to participate was obtained. Survey forms and envelopes were distributed by teachers to the students. Students were instructed to complete the questionnaires, place them in the provided envelopes, seal them, and return them to their teachers. The sealed envelopes were then forwarded to Oita University. A dataset combining baseline and follow-up survey data, organized by participant name and date of birth, was created. Thereafter, anonymized data were provided by Oita University to Nihon University after removing names, dates of birth, and school details to protect the personal information of the participants.

### Demographic characteristics and study design

Both the initial and follow-up surveys comprised identical paper-based questionnaires, which included sections on (1) demographic details, (2) commuting duration, (3) sleep-related factors, (4) lifestyle habits, and (5) school-life satisfaction. Demographic information collected included school grade, sex, age, and school identification number.

**Fig. 1:**
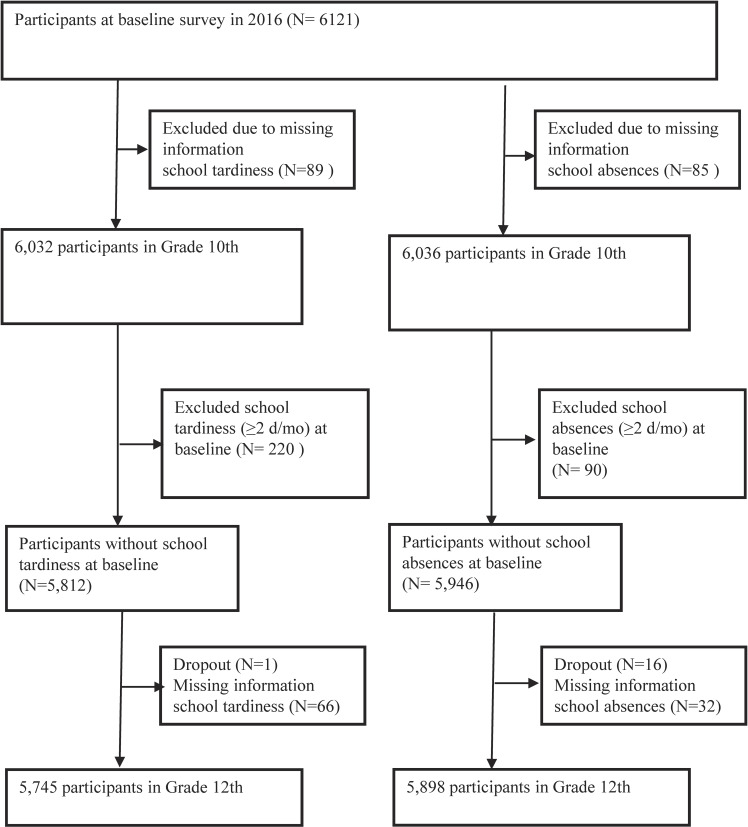
Flow chart of participant selection

Figure 1 presents a flowchart of participant selection. In the baseline survey, 6,121 10th-grade students participated. Those who did not answer the school attendance questions were excluded. Subsequently, we selected participants without school tardiness (n = 5,812: ≥2 days/month) and those without school absences (n = 5,946: ≥2 days/month) at baseline.

### School absenteeism

School absenteeism was assessed using two questions: (1) “In the past 30 days, have you ever been late for school due to difficulty awakening?” and (2) “In the past 30 days, have you ever missed school due to difficulty awakening?”

Response options for both included: none, 1 day, 2–4 days, 5–9 days, 10–19 days, and ≥20 days. A response of ≥2 days to either question was considered an affirmative answer, indicating a problem with tardiness or absences from school due to difficulty awakening.

### Commuting time

Participants were asked, “How long is your commuting time to school?” Response options were as follows: <30 min, ≥30 min to <1 h, ≥1 h to <2 h, and ≥2 h. Long commuters were defined as those with a commuting time of ≥1 h. Based on previous studies, commuting times were categorized as: <30 min, ≥30 min to <1 h, and ≥1 h [23].

### Covariates

Covariates included duration of sports extracurricular activities, study time outside school, internet usage time, sleep disturbance, napping time, and school satisfaction. Extracurricular activity duration and study time were categorized as: none, <1 h, 1–2 h, and ≥2 h.

Sleep disturbance was assessed using the Pittsburgh Sleep Quality Index (PSQI), a standardized, 18-item, self-administered instrument designed to retrospectively evaluate sleep habits and overall sleep quality over the previous month [27]. The Japanese version of the PSQI has demonstrated high reliability and validity [28]. The questions were divided into seven categories: subjective sleep quality, sleep latency, sleep duration, habitual sleep efficiency, sleep disturbances, use of sleep medication, and daytime dysfunction. Each component was rated on a scale of 0–3, where 0 indicated no risk, and ≥1 suggested potential risk. The cumulative score, ranging from 0 to 21, represented the global PSQI score. A cutoff score of 5.5 was used to distinguish between individuals with good sleep quality (≤5.5) and those with poor sleep quality (>5.5), with poor sleep quality indicating a higher risk for sleep issues.

Napping time was categorized as: none, <15 min, ≥15 min to <30 min, ≥30 min to <1 h, ≥1 h to <2 h, and ≥2 h. School-life satisfaction was classified into four categories: satisfied, somewhat satisfied, somewhat dissatisfied, and dissatisfied.

### Data analysis

Baseline characteristics were described using counts (n, %, 95% confidence intervals [CIs]) for categorical variables. Incidence rates of school tardiness and absences due to difficulty awakening were calculated, and Cox proportional hazards regression was used to explore the effects of commuting time on incident difficulty awakening, estimating hazard ratios (HRs) and their 95% CIs. Covariates included school, school type (public/private), sex, duration of sports extracurricular activities, study time outside school, internet usage time, sleep disturbance, napping time, and school-life satisfaction, based on previous studies [10, 13]. For absences, further analysis was conducted by including tardiness as an additional covariate. We confirmed that these covariates were weakly associated using Spearman’s correlation coefficient. In addition, cultural club activities were not used as a covariate because a strong inverse correlation was observed between them and athletic club activities.

Regarding sensitivity analyses, the Cox proportional hazards model was re-evaluated with an adjusted threshold, with the following changes: (1) limited to those who were satisfied with their school life and (2) specifically changing the criterion to ≥5 days/month of school tardiness or absences. All analyses were performed using Stata (version 17.0; StataCorp, College Station, TX, USA). Statistical significance was set at p < 0.05 (two-tailed).

## Results

Table 1 presents the baseline characteristics of participants without school attendance problems. Similar distributions were observed across each cohort. Approximately 50% of participants commuted to school in ≤30 min, used the internet <2 h/day, and did not experience sleep disturbances. Furthermore, approximately 80% of participants reported being satisfied or somewhat satisfied with their school lives. Compared with the participants without school absenteeism, those who developed school absenteeism were more likely to attend private schools, spend longer time on the internet, study for shorter durations, not participate in sports club activities, and report higher rates of sleep disturbances and school dissatisfaction (see Additional file 1 and Table 2).

**Table 1:** Baseline characteristics of participants

	**Participants ** **without school ** **tardiness** **(N = 5,812)**		**Participants ** **without school ** **absences** **(N = 5,946)**	

	**N**	**%**	**N**	**%**
Sex				
Men	2,899	49.9	2,971	50.0
Women	2,913	50.1	2,975	50.0
School type				
Public	4,502	77.5	4,502	75.7
Private	1,310	22.5	1,444	24.3
Commuting time				
<30 min	2,891	49.7	2,971	50.0
30–60 min	2,264	39.0	2,302	38.7
≥60 min	654	11.3	670	11.3
Unknown	3	0.1	3	0.1
Internet usage time				
<2 h	3,088	53.1	3,124	52.5
2–3 h	1,271	21.9	1,301	21.9
3–5 h	889	15.3	928	15.6
≥5 h	432	7.4	458	7.7
Unknown	132	2.3	135	2.3
Study time				
No	725	12.5	760	12.8
<1 h	1,633	28.1	1,667	28.0
1–2 h	1,996	34.3	2,032	34.2
≥2 h	1,353	23.3	1,380	23.2
Unknown	105	1.8	107	1.8
Sports club activity				
No	2,479	42.7	2,557	43.0
<1 h	147	2.5	148	2.5
1–2 h	430	7.4	437	7.3
≥2 h	2,725	46.9	2,770	46.6
Unknown	31	0.5	34	0.6
Sleep disturbance				
No	3,120	53.7	3,174	53.4
Yes	2,284	39.3	2,355	39.6
Unknown	408	7.0	417	7.0
Napping time				
No	1,790	30.8	1,814	30.5
<15 min	712	12.3	714	12.0
15–30 min	1,665	28.6	1,706	28.7
30–60 min	655	11.3	683	11.5
1–2 h	729	12.5	746	12.5
≥2 h	250	4.3	270	4.5
Unknown	11	0.2	13	0.2
School satisfaction				
Satisfied	2,179	37.5	2,215	37.3
Somewhat satisfied	2,496	42.9	2,548	42.9
Somewhat dissatisfied	807	13.9	845	14.2
Dissatisfied	325	5.6	333	5.6
Unknown	5	0.1	5	0.1

**Table 2:** Incidence of school absenteeism (tardiness and absences ≥2 days/month) due to difficulty awakening

	**Number**	**Incidence**	**Person-years** **/1,000**	**95% CI**
Tardiness				
≥2 days/month	5,812	224	19.27	16.91–21.97
≥5 days/month	5,987	88	7.35	5.96–9.06
Absence				
≥2 days/month	5,946	114	9.59	7.98–11.52
≥5 days/month	6,016	34	2.83	2.02–3.95

Abbreviations: CI = confidence interval.

Table 2 displays the incidence of school absenteeism due to difficulty awakening. Of the 5,812 participants without school tardiness due to difficulty awakening at baseline, 224 developed school tardiness during the 2-year follow-up (incidence rate: 19.27 per 1000 person-years [95% CI: 16.91–21.97]). Of the 5,946 participants without school absences due to difficulty awakening at baseline, 114 incurred school absences during follow-up (incidence rate: 9.59 per 1000 person-years [95% CI: 7.98–11.52]).

Table 3 presents the Cox proportional hazards analysis results, with school tardiness and absences (>2 days/month) due to difficulty awakening as the dependent variables. Regarding school tardiness, factors significantly associated with increased risk included: prolonged internet usage (2–3 h: HR = 1.69, 95% CI = 1.15–2.48; ≥5 h: HR = 1.87, 95% CI = 1.20–2.92), sleep disturbance (HR = 1.86, 95% CI = 1.38–2.51), long napping time (≥2 h: HR = 1.75, 95% CI = 1.05–2.94), being somewhat dissatisfied with school life (HR = 1.63, 95% CI = 1.06–2.49), and being dissatisfied with school life (HR = 2.84, 95% CI = 1.80–4.49).

**Table 3:** Factors associated with school absenteeism (tardiness and absences ≥2 days/month) due to difficulty awakening

	**Tardiness ≥2 days/month** **(N = 5,746)**			**Absences ≥2 days/month** **(N = 5,872)**		

	**HR**	**95% CI**	**p-value**	**HR**	**95% CI**	**p-value**
Sex (ref: men)						
Women	0.67	0.50–0.89	0.006	1.18	0.78–1.79	0.434
School type (ref: public)						
Private	1.15	0.69–1.89	0.594	1.02	0.46–2.27	0.955
Commuting time (ref: <30 min)						
30–60 min	0.86	0.63–1.18	0.352	1.20	0.75–1.93	0.450
≥60 min	1.15	0.75–1.77	0.523	2.93	1.75–4.91	<0.001
Internet usage time (ref: <2 h)						
2–3 h	1.69	1.15–2.48	0.008	1.40	0.79–2.50	0.252
3–5 h	1.33	0.86–2.04	0.197	1.34	0.72–2.48	0.359
≥5 h	1.87	1.20–2.92	0.006	2.13	1.17–3.89	0.014
Study time (ref: No)						
<1 h	0.61	0.40–0.92	0.017	0.78	0.43–1.42	0.415
1–2 h	0.68	0.44–1.05	0.084	1.23	0.70–2.16	0.477
≥2 h	0.59	0.39–0.90	0.014	0.66	0.35–1.25	0.204
Sports club activity (ref: No)						
<1 h	0.92	0.48–1.75	0.792	1.11	0.44–2.82	0.820
1–2 h	0.79	0.43–1.47	0.466	0.75	0.27–2.08	0.580
≥2 h	0.39	0.25–0.62	<0.001	0.71	0.35–1.42	0.332
Sleep disturbance (ref: No)						
Yes	1.86	1.38–2.51	<0.001	1.47	0.93–2.33	0.097
Napping time (ref: No)						
<15 min	0.53	0.27–1.04	0.066	2.43	1.02–5.80	0.045
15–30 min	0.85	0.57–1.27	0.436	1.57	0.80–3.08	0.192
30–60 min	1.01	0.62–1.64	0.974	1.84	0.86–3.91	0.115
1–2 h	1.26	0.82–1.94	0.292	1.28	0.62–2.67	0.503
≥2 h	1.75	1.05–2.94	0.033	1.65	0.73–3.76	0.232
School satisfaction (ref: Satisfied)						
Somewhat satisfied	1.15	0.79–1.68	0.461	1.12	0.62–2.03	0.701
Somewhat dissatisfied	1.63	1.06–2.49	0.025	2.37	1.32–4.29	0.004
Dissatisfied	2.84	1.80–4.49	<0.001	1.40	0.68–2.88	0.365
Tardiness (ref: 0 day)						
1 day				3.58	1.57–8.15	0.002
2–4 days				22.92	13.90–37.79	<0.001
≥5 days				27.22	14.61–50.72	<0.001

Adjusted for all the above variables and the school. Statistical significance is set at p < 0.05, with all tests being two-tailed. Missing values are excluded from the analyses.

HRs and p-values are calculated using the Cox proportional hazards model.

Abbreviations: CI = confidence interval; HR = hazard ratio.

Factors significantly associated with lower risk of school tardiness due to difficulty awakening included: being female (HR = 0.67, 95% CI = 0.50–0.89), study time (<1 h: HR = 0.61, 95% CI = 0.40–0.92; ≥2 h: HR = 0.59, 95% CI = 0.39–0.90), and participation in sports extracurricular activities (≥2 h: HR = 0.39, 95% CI = 0.25–0.62).

For school absences, the following factors were significantly associated with an increased risk due to difficulty awakening: long commuting time (>60 min: HR = 2.93, 95% CI = 1.75–4.91), prolonged internet usage (≥5 h: HR = 2.13, 95% CI = 1.17–3.89), being somewhat dissatisfied with school life (HR = 2.37, 95% CI = 1.32–4.29), and school tardiness (1 day: HR = 3.58, 95% CI = 1.57–8.15; 2–4 days: HR = 22.92, 95% CI = 13.90–37.79; ≥5 days: HR = 27.22, 95% CI = 14.61–50.72). In addition, as a sensitivity analysis limited to those who were satisfied with their school life, similar results were obtained using lateness or absence for two or more days as the dependent variable (see Additional file 3).

Table 4 presents the Cox proportional hazards analysis results using school tardiness and absences of >5 days/month due to difficulty awakening as dependent variables. The sensitivity analyses yielded results generally consistent with those in Table 3. However, regarding school absences, school satisfaction was not significantly associated with school absences.

**Table 4:** Factors associated with school absenteeism (tardiness and absences ≥5 days/month) due to difficulty awakening

	**Tardiness ≥5 days/month** **(N = 5,916)**			**Absences ≥5 days/month** **(N = 5,941)**		

	**HR**	**95% CI**	**p-value**	**HR**	**95% CI**	**p-value**
Sex (ref: men)						
Women	0.62	0.38–1.00	0.048	0.88	0.37–2.09	0.781
School type (ref: public)						
Private	0.39	0.17–0.89	0.026	0.99	0.19–5.24	0.988
Commuting time (ref: <30 min)						
30–60 min	0.60	0.34–1.06	0.077	1.86	0.67–5.21	0.235
≥60 min	1.21	0.63–2.32	0.572	4.68	1.64–13.36	0.004
Internet usage time (ref: <2 h)						
2–3 h	2.67	1.41–5.07	0.003	3.69	0.90–15.03	0.069
3–5 h	1.61	0.76–3.43	0.217	2.12	0.44–10.34	0.351
≥5 h	2.81	1.35–5.87	0.006	5.06	1.26–20.37	0.022
Study time (ref: No)						
<1 h	0.43	0.21–0.85	0.015	0.44	0.12–1.59	0.210
1–2 h	0.59	0.30–1.17	0.133	0.99	0.35–2.81	0.980
≥2 h	0.47	0.24–0.93	0.030	0.48	0.13–1.75	0.265
Sports club activity (ref: No)						
<1 h	0.96	0.38–2.43	0.925	1.92	0.45–8.14	0.375
1–2 h	0.18	0.02–1.31	0.090	1.46	0.30–7.05	0.639
≥2 h	0.41	0.20–0.87	0.019	0.35	0.04–2.78	0.319
Sleep disturbance (ref: No)						
Yes	1.87	1.13–3.08	0.014	0.90	0.36–2.25	0.828
Napping time (ref: No)						
<15 min	0.63	0.24–1.68	0.355	1.21	0.13–11.41	0.871
15–30 min	0.51	0.25–1.03	0.062	0.69	0.18–2.64	0.583
30–60 min	1.16	0.58–2.30	0.674	1.39	0.33–5.81	0.652
1–2 h	0.71	0.34–1.49	0.365	1.06	0.29–3.82	0.928
≥2 h	1.27	0.57–2.82	0.558	1.09	0.23–5.05	0.915
School satisfaction (ref: Satisfied)						
Somewhat satisfied	1.16	0.60–2.24	0.655	1.13	0.26–5.00	0.870
Somewhat dissatisfied	1.44	0.67–3.08	0.350	3.16	0.79–12.58	0.103
Dissatisfied	4.92	2.50–9.72	<0.001	2.26	0.54–9.53	0.267
Tardiness (ref: 0 day)						
1 day				6.65	1.23–35.85	0.028
2–4 days				19.91	5.60–70.78	<0.001
≥5 days				66.57	20.10–220.48	<0.001

Adjusted for all the above variables and the school. Statistical significance is set at p < 0.05, with all tests being two-tailed. Missing values are excluded from the analyses.

Abbreviations: CI = confidence interval; HR = hazard ratio. HRs and p-values are calculated using the Cox proportional hazards model.

## Discussion

To the best of our knowledge, this is the first longitudinal study to examine factors associated with incident school absenteeism due to difficulty awakening among Japanese adolescents. The principal findings were as follows: (1) a long commuting time was a risk factor for developing school absences, but not for tardiness due to difficulty awakening; (2) prolonged internet usage and poor school-life satisfaction were associated with an increased risk of both tardiness and absences; and (3) sleep disturbance and extended napping were risk factors and, greater study time and participation in sports extracurricular activities were protective factors for school tardiness. These results may inform the development of more effective strategies to prevent school absenteeism.

A long commuting time (>1 h) was a risk factor for school absences due to difficulty awakening. Several mechanisms may explain this association. First, longer school commutes have been associated with a heightened cortisol awakening response (CAR), particularly among individuals with an evening chronotype [29]. An elevated CAR is associated with increased stress and anxiety [29]. Moreover, extended commute times may reduce cortisol variability throughout the day, contributing to sustained stress levels and impaired hypothalamic–pituitary–adrenal axis recovery [29]. Second, long commutes themselves can be a considerable source of stress for adolescents [26]. Variability in commuting conditions, such as traffic congestion, delays, or disruptions caused by weather or accidents, can increase unpredictability and hinder timely arrival at school.

Adolescents with long commutes are required to wake up earlier to comply with school start times. Earlier wake times can increase the likelihood of daytime napping and may lead to earlier nap onset times on the same day [30]. Although napping does not always disrupt nocturnal sleep, prolonged or late-day naps may delay bedtime and reduce overall sleep duration, particularly on school days [30]. In the present study, short naps (≤30 min) appeared to be associated with a lower risk of tardiness due to difficulty awakening. Short naps are commonly recommended to prevent sleep inertia; however, the supporting evidence remains inconclusive and may vary depending on the context [31].

Consistent with previous studies [20, 21], sleep disturbances were associated with school tardiness due to difficulty awakening. Adolescents with orthostatic dysregulation (OD), a condition characterized by autonomic dysfunction, frequently experience difficulty awakening [32]. These individuals commonly exhibit reduced plasma renin activity and greater postural blood pressure reductions [33]. Difficulty awakening in adolescents with OD has been associated with delayed sleep–wake phase disorder [34].

Consistent with the results of previous research, our findings revealed that prolonged internet usage was a risk factor for school absenteeism due to difficulty awakening. Three consecutive cross-sectional studies conducted among Japanese adolescents showed that excessive screen time (>5 h/day) is strongly associated with various sleep problems, including shorter sleep duration and poorer sleep quality [35]. Similarly, sleep problems, such as insomnia and short sleep duration, have been associated with school absenteeism [36]. A 1-year longitudinal study of Swedish adolescents found that students with severe insomnia symptoms were approximately three times more likely to experience problematic absenteeism during follow-up than those with no or mild symptoms [37].

The findings of this study suggest that dissatisfaction with school is a risk factor for school absenteeism due to difficulty awakening, whereas study time and participation in extracurricular sports activities served as protective factors. A meta-analytic review identified various school-related issues as important risk factors for school absenteeism, including negative attitudes toward school [19]. Additionally, low academic achievement may increase the likelihood of school absenteeism [19]. Student-specific factors, such as completing assignments on time, are strong predictors of absenteeism severity [38]. However, in a sensitivity analysis, even when limited to satisfaction with school life, the factors associated with school absenteeism did not change, demonstrating the robustness of the results of this study.

Previous studies have shown that participation in sports and extracurricular activities has varying effects on school absenteeism; one study found that high school athletic participation was associated with a reduction in absenteeism, particularly unexcused absences, among boys [39]. Furthermore, involvement in extracurricular activities, specifically physical education and the arts, markedly reduced the likelihood of dropping out of high school, whereas participation in academic and vocational clubs had no such effect [40]. However, our results suggest that these protective effects diminish as absenteeism increases.

The strengths of this study include its longitudinal design, substantial sample size, consideration of potential confounding variables, and use of the PSQI for reliable assessments. In particular, this study included a large number of high schools within the region, thereby enhancing the validity of the results. However, this study had some limitations. First, because it was conducted within school environments, the rate of school absenteeism may have been underestimated, as we were unable to include adolescents who had already withdrawn from or discontinued their education. Second, this study focused on high school students from a specific prefecture in Japan. Consequently, caution is needed when generalizing these findings, as selection bias may be present. Future research should examine whether similar patterns are observed in other regions and countries to improve the external validity of the results. Third, despite efforts to adjust for potential confounding variables, we lacked data regarding certain familial and environmental factors, as well as modes of transportation, such as buses, railways, and walking. In particular, we lacked information on family discord, parental health history, divorce or separation, and aspects of the sleeping environment, including bedroom temperature and occupancy. Previous studies have demonstrated an association between these factors and school attendance challenges [41], suggesting that future research should include these variables in a more comprehensive analysis. Fourth, tardiness and absenteeism due to difficulty awakening are likely not entirely independent. However, this study showed that the risk and preventive factors for these two events differ in part, and that coping strategies differ depending on the stage. Finally, this study was conducted before the coronavirus disease 2019 (COVID-19) pandemic. After the pandemic, global school absenteeism rates increased [17, 42]. Therefore, this study does not address absenteeism issues specifically attributable to COVID-19.

In conclusion, the findings of the present study revealed that commuting times; lifestyle behaviors, including internet usage, study time outside school, participation in sports and extracurricular activities; sleep disturbances; and school satisfaction were associated with school absenteeism due to difficulty awakening. Thus, the results could inform the development of strategies to prevent school absenteeism. Educational institutions should consider measures to increase student satisfaction with school, such as providing learning support outside of class, supporting extracurricular activities, delaying start times, and improving the school environment. Conversely, it is crucial to establish an environment that enables educators to offer enhanced support to students’ daily lives. This can be achieved by implementing strategies to alleviate the workload on teachers, such as delegating club activities to local communities and conducting classes in collaboration with preparatory schools. Furthermore, collaboration between schools and parents is essential to promote sleep hygiene, limit excessive internet usage, and help students identify meaningful pursuits, including academic and athletic activities.

## Abbreviations

CI: confidence interval
HR: hazard ratio
PISA: Programme for International Student Assessment

## Supplementary information

**Additional file 1:** Baseline characteristics by school tardiness status (≥2 days/month) at follow-up.

**Additional file 2:** Baseline characteristics by school absences status (≥2 days/month) at follow-up.

**Additional file 3:** Factors associated with school absenteeism (tardiness and absences ≥2 days/month) due to difficulty awakening among school-satisfied adolescents.

## Declarations

### Availability of data and material

Data available on request from the authors.
