# Supplementary material for: Factors associated with school absenteeism due to difficulty awakening: a two-year prospective cohort study of Japanese adolescents
Source: Environ Health Prev Med. 2025 Nov 15;30:89. doi: 10.1265/ehpm.25-00290 (PMC12634215; doi:10.1265/ehpm.25-00290)
Supplement: Supplementary file 1 — Additional file 1: Baseline characteristics by school tardiness status (≥2 days/month) at follow-up. [file ehpm-30-089-s001.docx]

Additional file 1. Baseline characteristics by school tardiness status (≥2 days/month) at follow-up

|  | Participants without school tardiness at follow-up | | Participants with school tardiness at follow-up | |  |
| --- | --- | --- | --- | --- | --- |
|  |  |  |  |  |  |
|  | (N = 5,521) |  | (N = 224) |  |  |
| Sex |  |  |  |  | 0.150 |
| Men | 2,736 | 49.6 | 122 | 54.5 |  |
| Women | 2,785 | 50.4 | 102 | 45.5 |  |
| School type |  |  |  |  | 0.007 |
| Public | 4,297 | 77.8 | 157 | 70.1 |  |
| Private | 1,224 | 22.2 | 67 | 29.9 |  |
| Commuting time |  |  |  |  | 0.029 |
| < 30 min | 2729 | 49.4 | 131 | 58.5 |  |
| 30-60 min | 2165 | 39.2 | 71 | 31.7 |  |
| ≥ 60 min | 624 | 11.3 | 22 | 9.8 |  |
| Unknown | 3 | 0.1 | 0 | 0.0 |  |
| Internet usage time |  |  |  |  | < 0.001 |
| < 2 h | 2,976 | 53.9 | 86 | 38.4 |  |
| 2-3 h | 1,205 | 21.8 | 50 | 22.3 |  |
| 3-5 h | 827 | 15.0 | 52 | 23.2 |  |
| ≥ 5 h | 390 | 7.1 | 32 | 14.3 |  |
| Unknown | 123 | 2.2 | 4 | 1.8 |  |
| Study time |  |  |  |  | 0.002 |
| No | 665 | 12.0 | 43 | 19.2 |  |
| < 1 h | 1,543 | 27.9 | 69 | 30.8 |  |
| 1-2 h | 1,906 | 34.5 | 72 | 32.1 |  |
| ≥ 2 h | 1,310 | 23.7 | 36 | 16.1 |  |
| Unknown | 97 | 1.8 | 4 | 1.8 |  |
| Sports club activity |  |  |  |  | 0.002 |
| No | 2,331 | 42.2 | 116 | 51.8 |  |
| < 1 h | 137 | 2.5 | 8 | 3.6 |  |
| 1-2 h | 405 | 7.3 | 21 | 9.4 |  |
| ≥ 2 h | 2,619 | 47.4 | 77 | 34.4 |  |
| Unknown | 29 | 0.5 | 2 | 0.9 |  |
| Sleep disturbance |  |  |  |  | 0.011 |
| No | 2,979 | 54.0 | 100 | 44.6 |  |
| Yes | 2,158 | 39.1 | 104 | 46.4 |  |
| Unknown | 384 | 7.0 | 20 | 8.9 |  |
| Napping time |  |  |  |  | 0.049 |
| No | 1,708 | 30.9 | 58 | 25.9 |  |
| < 15 min | 671 | 12.2 | 35 | 15.6 |  |
| 15-30 min | 1,593 | 28.9 | 55 | 24.6 |  |
| 30-60 min | 622 | 11.3 | 23 | 10.3 |  |
| 1-2 h | 686 | 12.4 | 38 | 17.0 |  |
| ≥ 2 h | 232 | 4.2 | 14 | 6.3 |  |
| Unknown | 9 | 0.2 | 1 | 0.4 |  |
| School satisfaction |  |  |  |  | < 0.001 |
| Satisfied | 2,085 | 37.8 | 71 | 31.7 |  |
| Somewhat satisfied | 2,382 | 43.1 | 86 | 38.4 |  |
| Somewhat dissatisfied | 757 | 13.7 | 39 | 17.4 |  |
| Dissatisfied. | 292 | 5.3 | 28 | 12.5 |  |
| Unknown | 5 | 0.1 | 0 | 0.0 |  |
