# Supplementary material for: Factors associated with school absenteeism due to difficulty awakening: a two-year prospective cohort study of Japanese adolescents
Source: Environ Health Prev Med. 2025 Nov 15;30:89. doi: 10.1265/ehpm.25-00290 (PMC12634215; doi:10.1265/ehpm.25-00290)
Supplement: Supplementary file 2 — Additional file 2: Baseline characteristics by school absences status (≥2 days/month) at follow-up. [file ehpm-30-089-s002.docx]

Additional file 2. Baseline characteristics by school absences status (≥2 days/month) at follow-up

|  | Participants without school absences at follow-up | | Participants with school absences at follow-up | | |  |  |
| --- | --- | --- | --- | --- | --- | --- | --- |
|  |  |  |  |  |  |  |  |
|  | (N = 5,774) |  | (N = 114) | |  | p-value |  |
| Sex |  |  |  | |  | 0.879 |  |
| Men | 2,878 | 49.8 | | 56 | 49.1 |  |  |
| Women | 2,896 | 50.2 | | 58 | 50.9 |  |  |
| School type |  |  | |  |  | 0.012 |  |
| Public | 4,386 | 76.0 | | 75 | 65.8 |  |  |
| Private | 1,388 | 24.0 | | 39 | 34.2 |  |  |
| Commuting time |  |  | |  |  | 0.454 |  |
| < 30 min | 2878 | 49.8 | | 63 | 55.3 |  |  |
| 30-60 min | 2242 | 38.8 | | 38 | 33.3 |  |  |
| ≥ 60 min | 651 | 11.3 | | 13 | 11.4 |  |  |
| Unknown | 3 | 0.1 | | 0 | 0.0 |  |  |
| Internet usage time |  |  | |  |  | 0.001 |  |
| < 2 h | 3,058 | 53.0 | | 43 | 37.7 |  |  |
| 2-3 h | 1,259 | 21.8 | | 27 | 23.7 |  |  |
| 3-5 h | 893 | 15.5 | | 25 | 21.9 |  |  |
| ≥ 5 h | 434 | 7.5 | | 17 | 14.9 |  |  |
| Unknown | 130 | 2.3 | | 2 | 1.8 |  |  |
| Study time |  |  | |  |  | 0.014 |  |
| No | 724 | 12.5 | | 22 | 19.3 |  |  |
| < 1 h | 1,614 | 28.0 | | 36 | 31.6 |  |  |
| 1-2 h | 1,975 | 34.2 | | 41 | 36.0 |  |  |
| ≥ 2 h | 1,358 | 23.5 | | 14 | 12.3 |  |  |
| Unknown | 103 | 1.8 | | 1 | 0.9 |  |  |
| Sports club activity |  |  | |  |  | 0.005 |  |
| No | 2,469 | 42.8 | | 59 | 51.8 |  |  |
| < 1 h | 141 | 2.4 | | 6 | 5.3 |  |  |
| 1-2 h | 422 | 7.3 | | 12 | 10.5 |  |  |
| ≥ 2 h | 2,710 | 46.9 | | 36 | 31.6 |  |  |
| Unknown | 32 | 0.6 | | 1 | 0.9 |  |  |
| Sleep disturbance |  |  | |  |  | 0.012 |  |
| No | 3,091 | 53.5 | | 47 | 41.2 |  |  |
| Yes | 2,279 | 39.5 | | 57 | 50.0 |  |  |
| Unknown | 404 | 7.0 | | 10 | 8.8 |  |  |
| Napping time |  |  | |  |  | 0.193 |  |
| No | 1,759 | 30.5 | | 31 | 27.2 |  |  |
| < 15 min | 696 | 12.1 | | 13 | 11.4 |  |  |
| 15-30 min | 1,670 | 28.9 | | 25 | 21.9 |  |  |
| 30-60 min | 657 | 11.4 | | 17 | 14.9 |  |  |
| 1-2 h | 721 | 12.5 | | 20 | 17.5 |  |  |
| ≥ 2 h | 259 | 4.5 | | 8 | 7.0 |  |  |
| Unknown | 12 | 0.2 | | 0 | 0.0 |  |  |
| School satisfaction | |  | |  |  | < 0.001 |  |
| Satisfied | 2,162 | 37.4 | | 36 | 31.6 |  |  |
| Somewhat satisfied | 2,475 | 42.9 | | 44 | 38.6 |  |  |
| Somewhat dissatisfied | 818 | 14.2 | | 20 | 17.5 |  |  |
| Dissatisfied. | 314 | 5.4 | | 14 | 12.3 |  |  |
| Unknown | 5 | 0.1 | | 0 | 0.0 |  |  |
|  | |  |  | |  |  |  |
