# Supplementary material for: Factors associated with school absenteeism due to difficulty awakening: a two-year prospective cohort study of Japanese adolescents
Source: Environ Health Prev Med. 2025 Nov 15;30:89. doi: 10.1265/ehpm.25-00290 (PMC12634215; doi:10.1265/ehpm.25-00290)
Supplement: Supplementary file 3 — Additional file 3: Factors associated with school absenteeism (tardiness and absences ≥2 days/month) due to difficulty awakening among school-satisfied adolescents. [file ehpm-30-089-s003.docx]

Additional file 3. Factors associated with school absenteeism (tardiness and absences ≥2 days/month) due to difficulty awakening among school-satisfied adolescents

|  | Tardiness ≥ 2 days/month (N=5,122) | | | | |  | Absences ≥ 2 days/month (N=5,219) | | | | |
| --- | --- | --- | --- | --- | --- | --- | --- | --- | --- | --- | --- |
|  | HR | 95% CI | | | p-value |  | HR | 95% CI | | | p-value |
| Sex (ref: men) |  |  |  |  |  |  |  |  |  |  |  |
| Women | 0.67 | 0.50 | – | 0.89 | 0.006 |  | 1.57 | 0.84 | – | 2.94 | 0.159 |
| School type (ref: public) | |  |  |  |  |  |  |  |  |  |  |
| Private | 1.15 | 0.69 | – | 1.89 | 0.594 |  | 0.63 | 0.21 | – | 1.87 | 0.404 |
| Commuting time (ref: < 30 min) | | |  |  |  |  |  |  |  |  |  |
| 30–60 min | 0.86 | 0.63 | – | 1.18 | 0.352 |  | 1.30 | 0.68 | – | 2.50 | 0.426 |
| ≥ 60 min | 1.15 | 0.75 | – | 1.77 | 0.523 |  | 2.75 | 1.21 | – | 6.26 | 0.016 |
| Internet usage time (ref: < 2 h) | | |  |  |  |  |  |  |  |  |  |
| 2–3 h | 1.69 | 1.15 | – | 2.48 | 0.008 |  | 1.88 | 0.81 | – | 4.37 | 0.144 |
| 3–5 h | 1.33 | 0.86 | – | 2.04 | 0.197 |  | 1.89 | 0.76 | – | 4.72 | 0.171 |
| ≥ 5 h | 1.87 | 1.20 | – | 2.92 | 0.006 |  | 2.71 | 1.10 | – | 6.68 | 0.031 |
| Study time (ref: No) | |  |  |  |  |  |  |  |  |  |  |
| < 1 h | 0.61 | 0.40 | – | 0.92 | 0.017 |  | 0.63 | 0.26 | – | 1.52 | 0.302 |
| 1–2 h | 0.68 | 0.44 | – | 1.05 | 0.084 |  | 0.80 | 0.32 | – | 2.03 | 0.644 |
| ≥ 2 h | 0.59 | 0.39 | – | 0.90 | 0.014 |  | 0.76 | 0.31 | – | 1.84 | 0.540 |
| Sports club activity (ref: No) | | |  |  |  |  |  |  |  |  |  |
| < 1 h | 0.92 | 0.48 | – | 1.75 | 0.792 |  | 0.44 | 0.06 | – | 3.31 | 0.429 |
| 1–2 h | 0.79 | 0.43 | – | 1.47 | 0.466 |  | 0.38 | 0.05 | – | 2.80 | 0.342 |
| ≥ 2 h | 0.39 | 0.25 | – | 0.62 | < 0.001 |  | 0.38 | 0.13 | – | 1.12 | 0.080 |
| Sleep disturbance (ref: No) | | |  |  |  |  |  |  |  |  |  |
| Yes | 1.86 | 1.38 | – | 2.51 | < 0.001 |  | 1.55 | 0.81 | – | 2.98 | 0.183 |
| Napping time (ref: No) | |  |  |  |  |  |  |  |  |  |  |
| < 15 min | 0.53 | 0.27 | – | 1.04 | 0.066 |  | 2.68 | 0.75 | – | 9.49 | 0.127 |
| 15–30 min | 0.85 | 0.57 | – | 1.27 | 0.436 |  | 2.14 | 0.75 | – | 6.16 | 0.157 |
| 30–60 min | 1.01 | 0.62 | – | 1.64 | 0.974 |  | 2.45 | 0.78 | – | 7.73 | 0.127 |
| 1–2 h | 1.26 | 0.82 | – | 1.94 | 0.292 |  | 1.85 | 0.57 | – | 5.99 | 0.306 |
| ≥ 2 h | 1.75 | 1.05 | – | 2.94 | 0.033 |  | 2.25 | 0.63 | – | 7.97 | 0.210 |
| Tardiness (ref: 0 day) | |  |  |  |  |  |  |  |  |  |  |
| 1 day |  |  |  |  |  |  | 3.58 | 1.57 | – | 8.15 | 0.002 |
| 2-4 days |  |  |  |  |  |  | 22.92 | 13.90 | – | 37.79 | < 0.001 |
| ≥ 5 days |  |  |  |  |  |  | 27.22 | 14.61 | – | 50.72 | < 0.001 |

Adjusted for all the above variables and the school. Statistical significance is set at p<0.05, with all tests being two-tailed. Missing values were excluded from the analyses.

HRs and p-values are calculated using the Cox proportional hazards model.

Abbreviations: CI=confidence interval; HR=hazard ratio.
